# How Glucose Is Made

**Published:** 1889-09

**Authors:** 


### HOW GLUCOSE IS MADE.

The process of making glucose will be best understood, says the American Analyst, by following the corn from the time it enters the factory until it runs out at spigot, a clear, odorless liquid. The shell corn is first soaked for several days in water to soften the hull and prepare it for the cracking process. The softened corn is conveyed by elevators to one of the highest stories of the factory and shoveled into large hoppers, from which it passes into mills that merely crack the grains without reducing them at once to fine meal. The cracked grain is then conducted to a large tank filled with rinsing water. The hulls of the corn float at the top of the water, the germs sinks to the bottom, and the portions of the grain containing the starch, becoming gradually reduced to flour by friction are held in solution in the water. By an ingenious process both the hulls and the germs are removed and the flour part now held in solution coutains nothing but starch and gluten.

This liquid is then made to flow over a series of tables, representing several acers in area, and the difference in the specific gravity of the two substances causes the gluten and starch to separate without the use of chemicals. The gluten is of a golden-yellow color and the starch snow white. By the time the gluten has been completely eliminated the starch assumes a plastic form and is collected from the separating tables by wheelbarrowsful and taken to a drying-room where it is prepared as the starch of commerce or is placed in a chemical apparatus to be converted into glucose.

